# Evaluation of Zein Nanoparticles as Delivery Agents of SARS-CoV-2 Antigens

**DOI:** 10.3390/vaccines13020139

**Published:** 2025-01-28

**Authors:** Verónica Araceli Márquez-Escobar, María José Alonso-Cerda, Sergio Rosales-Mendoza, María de Lourdes Betancourt-Mendiola

**Affiliations:** 1 Biotechnology Section, Center for Research in Health Science and Biomedicine, Autonomous University of San Luis Potosí, Av. Sierra Leona 550, Lomas de San Luis, San Luis Potosí 78210, Mexico; vero_marquez_333@hotmail.com (V.A.M.-E.); a206367@uaslp.mx (M.J.A.-C.); 2 Recombinant Biopharmaceuticals Laboratory, School of Chemical Sciences, Autonomous University of San Luis Potosí, Manuel Nava 6, Av. Dr. Manuel Nava, San Luis Potosí 78210, Mexico

**Keywords:** organic nanoparticle, adjuvanticity, humoral response, polydispersity, COVID-19

## Abstract

Background/Objectives: Nanovaccines have significant potential to enhance immunization strategies by improving efficacy, safety, and cost-effectiveness. In particular, organic nanoparticles hold promise for the generation of low-cost nanovaccines obtained by environmentally friendly methods. In this study, the feasibility of using zein nanoparticles (NPs) as carriers for an antigenic peptide (p30) and the receptor binding domain (RBD) from SARS-CoV-2 spike protein was explored. Methods: A synthesis method for zein NPs was established by combining previously reported techniques, and the resulting NPs were characterized in terms of morphology, particle size, polydispersity index (PDI), surface charge, and colloidal stability using dynamic light scattering (DLS) and transmission electron microscopy (TEM). Tween 20 was employed as a surfactant to enhance particle stability and prevent aggregation. Results: The zein NPs were deemed safe based on an in vitro cytotoxicity assay using Vero cells. Immunogenicity assessments demonstrated that zein NPs:p30 and zein NPs:RBD induced IgG responses in test mice, whose magnitude was comparable to those achieved with alum as an adjuvant. Conclusions: These findings support the use of zein NPs as promising vaccine delivery vehicles with adjuvant effects due to their ease and environmentally friendly synthesis, high stability, and low cost.

## 1. Introduction

In recent years, the development of biocompatible nanoparticles (NPs) has gained significant attention across various scientific fields, particularly in biomedicine. The use of such nanomaterials as antigen delivery systems has shown great promise in vaccinology, leading to the development of new vaccines [1], which are highly effective due to enhanced stability, antigen delivery, and immunogenicity. The efficacy of nanovaccines relies in part on the ability of NPs to facilitate immunogen uptake by antigen-presenting cells (APCs), leading to enhanced antigen presentation and stronger immune responses. Additionally, NPs may induce the production of proinflammatory cytokines, promoting immune cell recruitment and activation. For instance, nanovaccines based on lipid NPs for RNA delivery have significantly impacted the reduction of incidence, hospitalization, and mortality associated with COVID-19 [2,3].

Nanoparticles are classified as organic, inorganic, and carbon-based categories based on their chemical composition [4,5]. Inorganic NPs are derived from metals, metal oxides, semiconductors, and ceramics, offering advantages such as enhanced stability and smaller size compared to organic NPs. Conversely, organic NPs are composed of proteins, lipids, polymers, carbohydrates, among other organic compounds. Organic NPs are generally biodegradable and biocompatible, making them highly attractive for biomedical applications [6]. The adjuvant effects of organic NPs have also been demonstrated; for example, chitosan-based organic NPs loaded with measles antigen induced a robust humoral immune response following oral immunization in mice by stimulating IgA-secreting cells in the gut-associated lymphoid tissue (GALT) [7]. Additionally, a poly(lactic-co-glycolic acid) (PLGA)-based nanovaccine utilizing the inactivated polio vaccine and various cationic groups elicited neutralizing responses comparable to those observed in humans [8]. Furthermore, liposomes have intrinsic adjuvant properties due to their phospholipid content, as evidenced by the development of Epaxal^®^ and Inflexal^®^, which protect against hepatitis A and seasonal influenza, respectively [9,10]. These formulations have been shown to enhance both humoral and cellular immune responses induced by various antigens [11].

Proteins can also be utilized for synthesizing attractive NPs in vaccine design due to their biocompatibility and, in some cases, low cost. They exhibit enhanced affinity for biomolecules through several molecular interactions, including hydrogen bonds and hydrophobic or π–π interactions [12]. Protein-based NPs are capable of providing sustained release of active compounds due to their hydrophobic nature [13].

Among the proteins with the ability to form NPs, zein—a prolamine protein derived from maize seeds—has emerged as a promising candidate for NP synthesis due to its remarkable biocompatibility, low cost, and relatively straightforward surface modification [14]. Based on its intrinsic characteristics, zein has been classified as generally recognized as safe (GRAS) by the Food Drug Administration (FDA) [15]. Zein NPs have been employed as versatile platforms for encapsulating bioactive compounds such as vitamin D3 [16], folic acid [17], doxorubicin [18], and rhodamine B [19], demonstrating their broad range of biomedical applications. The surface charge of zein is pH-dependent, thus appropriately designed pH changes can promote electrostatic interactions with hydrophilic molecules possessing opposite charges [13]. The synthesis of zein NPs has been achieved through various strategies including antisolvent precipitation [20,21], pH- or heat-induced antisolvent precipitation [22,23,24], and antisolvent coprecipitation [25,26].

Given the attributes of zein NPs, their potential application in vaccine development deserves attention. However, an analysis of the current literature reveals that their use in this field has not been systematically explored. Nanovaccines based on zein NPs could result in low-cost vaccines, which is crucial for widespread immunization efforts, especially in resource-limited settings. The low cost of zein NP-based vaccines is supported by their source—corn seeds—and the simplicity of the methods used for their synthesis, which do not require complex purification steps or costly and/or toxic reagents. Additionally, the safety profile of zein NPs is a critical advantage for the development of human vaccines against both infectious and non-communicable diseases. These attributes are particularly notable when compared to existing nanoparticle-based vaccine strategies.

This study aimed at establishing and optimizing a synthesis method for zein NPs by adjusting factors such as zein concentration, solvent choice, and purification conditions to improve particle size, morphology, and stability. Additionally, the ability of zein NPs to form stable composites and their potential to enhance the induction of humoral immune responses in mice were evaluated by comparing their magnitude with those induced by a conventional adjuvant. The novelty of this research lies in its systematic approach for zein NPs synthesis, an area that has not been extensively explored. By focusing on stable conjugates formation and assessing immunogenic potential against a traditional adjuvant, the study provides new insights into zein NPs as effective vaccine delivery vehicles. Furthermore, the environmentally friendly and low-cost production method highlights their practical applications in vaccine development.

## 2. Materials and Methods

### 2.1. Reagents

Zein, bovine serum albumin (BSA), and Tween 20 were obtained from Sigma-Aldrich (St Louis, MO, USA). All other solvents and chemicals used were of analytical grade or equivalent.

### 2.2. Zein NP Synthesis

Zein NPs were synthesized employing the nanoprecipitation method [27]. Briefly, a zein solution (0.5 mg/mL) in 80% (*v*/*v*) ethanol–water was stirred until complete dissolution. The solution was then filtered through a cellulose acetate filter with a pore diameter of 0.22 µm to remove any large particles. The filtered zein solution was added dropwise to double-filtered water at a 5:1 ratio (water:zein) with a total flow rate of 1 mL/min. This process was carried out under vigorous stirring at 1000 rpm. To prevent aggregation, 0.022% of Tween 20 was added as a surfactant. The suspension pH was adjusted to 10 to facilitate resuspension after centrifugation. Ethanol was removed by centrifugation at 10,000× *g* for 10 min at room temperature. Finally, the zein NPs were resuspended in the appropriate buffer and stored at 4 °C in the dark.

### 2.3. Zein NPs:BSA Synthesis

The adsorption process involved dispersing zein NPs in the adsorption buffer (20 mM Tris, pH 5.5). Three different mass ratios were evaluated to formulate stable NPs with the antigen. Zein NPs (50 µg/mL) and bovine serum albumin (BSA) (100, 50, and 25 µg/mL), both in 20 mM Tris (pH 5.5), were mixed to achieve zein NPs/BSA mass ratios of 1:2, 1:1, and 1:0.5. The adsorption process was conducted in a rotating mixer at room temperature for 1 h at 30 rpm. The resulting zein NPs:BSA were centrifuged at 10,000× *g* for 10 min and washed twice. The supernatant was collected to quantify the non-bound antigen, and the pellet was resuspended in 20 mM Tris (pH 7.4) as a maintenance buffer. The non-bound antigens in the supernatant were quantified using UV-Vis spectroscopy.

### 2.4. Zein NPs:p30 Synthesis

The p30 peptide, with the sequence DSFKEELDKYFKNHTS, is derived from the spike protein of SARS-CoV-2 (residues S_1146–1161_) and was synthesized by GenScript (Piscataway, NJ, USA), with a reported purity > 95%. The peptide was dissolved in 20 mM Tris buffer (pH 5.8) at a concentration of 2 µg/µL. Adsorption of p30 onto zein NPs was carried out in 20 mM Tris buffer (pH 5.8) as the adsorption buffer. For the adsorption process, zein NPs (25 µg/mL) and p30 (50 µg/mL) were mixed at room temperature for 1 h with continuous rotation (30 rpm). The resulting zein NPs:p30 were centrifuged at 10,000× *g* for 10 min and washed twice. The supernatant was collected to quantify the non-bound antigen, and the pellet was resuspended in 20 mM Tris buffer (pH 7.4). The amount of the non-bound antigen in the supernatant was quantified using UV-Vis spectroscopy.

### 2.5. Zein NPs:RBD Synthesis

Recombinant SARS-CoV-2 RBD protein was purchased from Sino Biological (Beijing, China, Cat: 40592-V08H121, purity > 95%, as determined by SDS-PAGE and SEC-HPLC). The RBD antigen, originally in PBS (100 µg/mL), was dialyzed against 20 mM Tris (pH 5.5) using a cellulose membrane with a molecular weight cutoff (MWCO) of 3.5 kDa. The RBD antigen was incorporated to zein NPs by incubating (10.4 µg/mL) and RBD (20.8 µg/mL) at room temperature for 1 h with continuous rotation at 30 rpm; this process was carried out in 20 mM Tris (pH 5.5). The resulting Zein NPs:RBD were centrifuged at 10,000× *g* for 10 min and, after discarding the supernatant, the pellet was resuspended in 480 µL of 20 mM Tris (pH 7.4).

### 2.6. Adsorption Efficacy

The adsorption efficiency (AE) of BSA, RBD, or p30 on zein NPs was calculated applying an indirect method, expressed by the following formula:

$$AE=\frac{Initial\;amount\;of\;antigen-Amount\;of\;antigen\;in\;the\;supernatant}{Initial\;amount\;of\;antigen}\times 100$$

The AE of the target antigen on zein NPs was determined by the difference between the initial amount of antigen added during the adsorption (total amount) and the remaining antigen quantified in the supernatant after centrifugation (amount in the supernatant). A standard curve for each antigen was generated to quantify the amount of antigen in the samples. The absorbance of each sample was measured using UV-Vis spectrophotometry with an Eppendorf Biophotometer Plus (Eppendorf, Hamburg, Germany) at 280 nm.

### 2.7. TEM Morphological Analysis

The morphological structures of zein NPs and their derivatives were analyzed by TEM using a JEOL JEM-1200EX^®^ transmission electron microscope (JEOL, Tokyo, Japan), operating at an accelerating voltage of 80 kV. Each sample was diluted at 1:30 in double-filtered water. A 5 µL aliquot of each sample was applied to a lacey Formvar/carbon grid (300-mesh size) and air-dried overnight prior to imaging.

### 2.8. Determination of Hydrodynamic Diameter, Size Distribution, and Zeta Potential

The mean size of the NPs, along with their size distribution, was determined by measuring the hydrodynamic diameter and PDI using DLS with a Nano Zetasizer (Malvern Instruments Ltd., Malvern, UK). The zeta potential of the different samples was measured using a He-Ne laser (633 nm) with a folding capillary cuvette (Zetasizer, Malvern Instruments Ltd., Malvern, UK). All measurements were conducted at 25 ± 1 °C in triplicate. Prior to measurement, all samples were diluted 1:10 (*v*/*v*) with ultrapure water.

### 2.9. Cytotoxicity Assessment

The cytotoxicity of zein NPs was evaluated using the resazurin assay with the Vero cell line. Cells were cultured in DMEM (Corning Inc., Corning, New York, USA), supplemented with ampicillin/streptomycin (Thermo Fisher Scientific, Waltham, MA, USA) and 10% heat-inactivated fetal bovine serum (Gibco^®^ Thermo Fisher Scientific, Waltham, MA, USA) at 37 °C in a 5% CO_2_ atmosphere until they reached confluence. The cells were seeded in duplicate at a density of 1 × 10^5^ cells per well in 24-well plates. Following seeding, the cells were incubated with zein NPs at varying concentrations (12, 25, 50, and 100 µg/mL) for 24 h at 37 °C in a 5% CO_2_ atmosphere. As viability control, cells were treated with the vehicle alone (20 mM Tris, pH 7.4), and as a negative control, cells were exposed to 40 mM hydrogen peroxide (H_2_O_2_). After incubation, the medium was replaced, and the cells were treated with 30 mg/mL resazurin for 4 h. Fluorescence (560 nm/590 nm) was measured using a FlexStation II scanning fluorimeter (Molecular Devices LLC, San Jose, CA, USA) with SoftMax Pro software (Version 4.8).

### 2.10. Immunogenicity Assessment of Zein NPs:p30 and Zein NPs:RBD

The protocols for assessing the immunogenicity of zein NPs:p30 and zein NPs:RBD were conducted in accordance with the Guide for the Care and Use of Laboratory Animals by the National Institutes of Health (NIH, Bethesda, MD, USA) and were approved by the Institutional Ethics Committee of the Chemical Faculty at the University of San Luis Potosí, Mexico, under Registration Number: CEID-2020-07-R1. Experimental groups of BALB/c mice (*n* = 4, 8 weeks old) were established to perform a dose–response experiment. Mice were immunized via the subcutaneous (s.c.) route on days 1, 14, and 28 with one of the following formulations: (1) 100 µL of 20 mM Tris, pH 7.4 (vehicle); (2) zein NPs:p30 5 µg; (3) zein NPs:p30 10 µg; or (4) 10 µg of p30 peptide and aluminum hydroxide as an adjuvant. For the assessment related to the RBD antigen, mice experimental groups received one of the following treatments: (1) 100 µL of PBS, pH 7.4 (vehicle); (2) zein NPs:RBD 1 µg; or (3) 1 µg of RBD antigen with aluminum hydroxide as an adjuvant. Blood samples were collected by tail puncture on days 14, 28, and 42. The blood samples were centrifuged at 6000 rpm for 10 min, and the sera were separated and stored at −20 °C until further analysis by ELISA.

### 2.11. Antibody Determinations

Antibody levels of total IgG, IgG1, IgG2a, and IgM were determined by indirect ELISA using the p30 peptide, spike protein, or RBD protein from SARS-CoV-2 as target antigens. Polystyrene 96-well plates were coated overnight at 4 °C with the target antigen (200 ng per well) diluted in 0.2 M carbonate buffer (15 mM Na_2_CO_3_ + 35 mM NaHCO_3_, pH 9.6). Three washes with PBST (PBS 1× + 0.05% Tween 20) were performed between each step. The plates were blocked with fat-free milk (a 5% solution prepared in PBS) at room temperature for 2 h. Wells were added with sera and subjected to overnight incubation at 4 °C. The plates were subsequently incubated for 2 h at room temperature with peroxidase-conjugated anti-mouse IgG1, IgG2a, or IgM secondary antibody (1:2000). The colorimetric detection was performed by using ABTS (0.3 mg/L ABTS in 0.6 mM 2,2′-azino-bis(3-ethylbenzothiazoline-6-sulfonic acid, 0.1 M citric acid, and 1 mM H_2_O_2_, pH 4.35) under a 1 h-incubation at 25 °C. Optical density values were determined at 405 nm in a Multiskan^®^ FC microplate photometer (Thermo Fisher Scientific, Waltham, Massachusetts, United States of America).

### 2.12. Statistical Analyses

Means and standard errors were calculated for each dataset. For the data on humoral responses, one-way ANOVA was performed to determine significant differences among groups, followed by Fisher’s LSD multiple comparisons test. All data were analyzed and graphed using GraphPad Prism v6 (GraphPad Software, San Diego, CA, USA).

## 3. Results

### 3.1. Synthesis of Zein NPs

**Figure 1:**
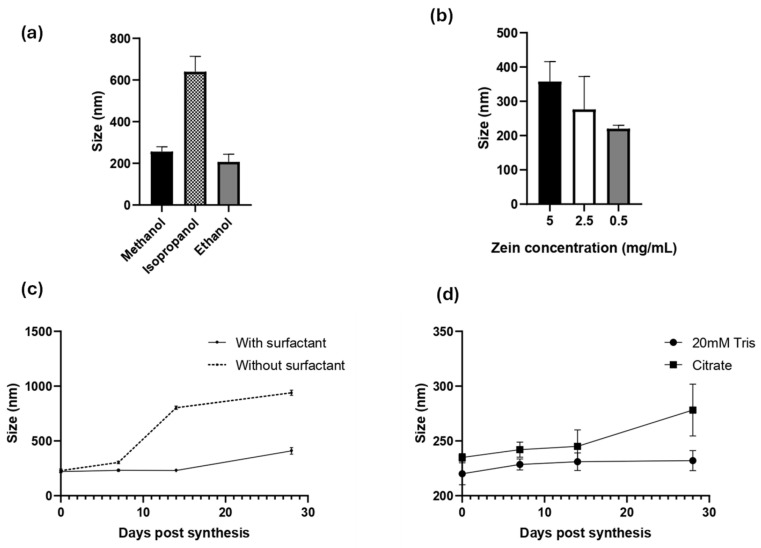
Characterization of zein NP synthesis by DLS: the effects of (**a**) solvent, (**b**) inicial zein concentration effect, (**c**) surfactant addition, and (**d**) resuspension buffer are presented. Mean values ± SD are presented.

Zein NPs were successfully prepared by a simple nanoprecipitation method. Several experimental conditions were evaluated to obtain an accurate and reproducible method. Since zein is soluble in alcoholic solutions, the effect of the solvent was evaluated in either 80% (*v*/*v*) methanol, ethanol, or isopropanol (Figure 1a). DLS was used to monitor the hydrodynamic diameter (d_H_), PDI, and zeta potential of the obtained zein NPs. In terms of diameter and PDI, ethanol was the best solvent to synthetize the zein NPs, reaching 207 ± 36.7 nm, a low PDI of 0.188 ± 0.010, and a negative zeta potential of −24.8 ± 10.4 (Table S1a).

Once the solvent was selected, the effect of the zein concentration was evaluated using the following levels: 5, 2.5, or 0.5 mg/mL (Figure 1b, Table S1b). This analysis revealed that the higher concentration of zein leads to the largest particle size, whereas lower concentrations led to a lower size and PDI values. The use of 0.5 mg/mL zein allowed the synthesis of the smallest zein NPs, with a mean diameter of 220 ± 10 nm, PDI = 0.13 ± 0.15, and zeta potential = −8.9 ± 0.89.

To improve their physical stability and prevent aggregation, a surfactant is often added during synthesis of organic NPs. Due to its intrinsic hydrophobicity, zein NPs tend to lose physical stability and aggregate. To address this issue, Tween 20, a widely used non-ionic polyoxyethylene surfactant, was used in this study. Tween 20 was added at a concentration of 0.22 g/L in the aqueous phase during nanoprecipitation (Figure 1c, Table S1c), and the physical stability of the zein NPs was followed through the time by DLS (Figure 1c). Indeed, when zein NPs were synthetized in the presence of Tween 20, the size at day 0 was 220 ± 10 nm, whereas at the end of the assay on day 28 the size slightly increased to 232 ± 9.2 nm. In contrast, the size of zein NPs synthetized in absence of Tween 20 was 228.8 ± 15 nm and increased over the time of the assay, reaching 802.8 ± 15 nm at 14 days-post-synthesis, indicating the formation of aggregates (Table S1c). These results suggest that the addition of the surfactant improved the physical stability of zein NPs.

Considering that a drug formulation needs to be stable at physiological pH, two different buffer solutions were evaluated as vehicles of zein NPs: 20 mM Tris, pH 7.4 and citrate buffer, pH 7.4. The stability of zein NPs in the test buffers was evaluated at different post-synthesis time points (Figure 1d). It was demonstrated that 20 mM Tris buffer allowed the particles to maintain their size (mean value: 232 ± 9.2 nm), while citrate buffer led to an increased size by the end of the analysis (mean value: 278.2 ± 23.7 nm) at day 28 (Table S1d).

In summary, the method established for zein NP synthesis and stabilization is based on the following parameters: 80% (*v*/*v*) ethanol for dissolution of zein, 0.5 mg/mL zein, 0.022% of Tween 20 as surfactant, and 20 mM Tris pH 7.4 as the dispersion buffer. Using the optimized method, the average diameter of the zein NPs was found to be 220 ± 10 nm, with PDI values of 0.13 ± 0.15, indicating monodisperse NPs and a negative zeta potential (−8.9 ± 0.89).

### 3.2. Preparation of Zein NPs:BSA

To determinate the capability of the zein NPs to load antigens by physical adsorption, BSA was used at first as a model antigen. BSA was incorporated onto zein NPs at 3 different mass ratios of zein NPs:BSA (1:0.5, 1:1, and 1:2) (*w*:*w*) by dispersion of BSA on the zein NPs solution in 20 mM Tris, pH 7.4. The size, PDI, and zeta potential of each adsorption condition were measured by DLS, and the obtained data are presented in Table 1. The maximum AE was found at the 1:2 mass ratio (zein NPs:BSA), with a 63.8% value. Interestingly, an approximately 2-fold increase was observed when BSA was incorporated (size of bare zein NPs = 220 ± 10 nm versus zein NPs:BSA : 424.3 ± 36.3 nm). Furthermore, to optimize the AE, the influence of pH variation during the adsorption process was evaluated, considering the pI of both zein (6.2) and BSA (4.7) [28,29]. Looking for conditions to favor electrostatic interactions between zein NPs and BSA, a pH value of 5.5 was selected since in this condition, zein NPs are positively charged, whereas BSA are negatively charged, thus favoring electrostatic interactions

Under the improved adsorption conditions, the adsorption of BSA onto zein NPs was evaluated only for the 1:2 mass ratio (zein NPs:BSA). As expected, the AE of the zein NPs increased from 63.8% to 69.1%, and the size of the zein NPs:BSA conjugates was reduced from 424.3 ± 36.3 nm to 329.1 ± 2.1 nm, confirming that pH is a critical factor to favoring the absorption of BSA onto zein NPs.

### 3.3. Preparation of Zein NPs:p30

**Figure 2:**
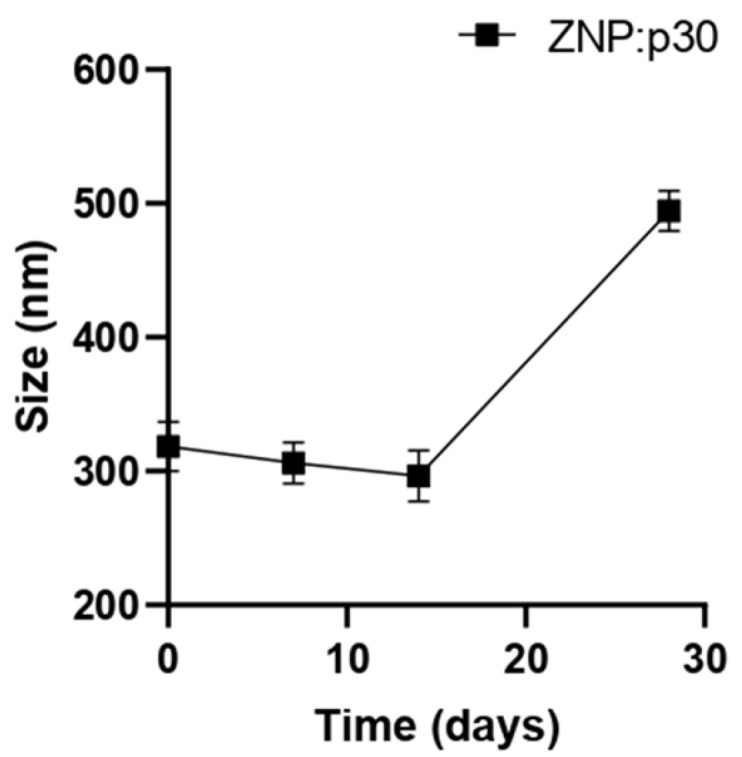
Stability of zein NPs:p30 (*n* = 3) at different time points (0, 7, 14, and 28 days). Samples were stored at 4 °C in the dark. Mean values ± SD are presented.

To evaluate the potential of zein NPs to serve as antigen carriers able to enhance immunogenicity, a target peptide (p30) was incorporated into zein NPs. The p30 peptide is derived from the SARS-CoV-2 spike protein. The adsorption of p30 onto zein NPs was performed with a mass ratio of 1:2. Taking into consideration that the theorical isoelectric point of p30 is 5.48 and the point of zero charge (PZC) for zein is 6.2, the adsorption buffer used was 20 mM Tris at pH 5.8. At this pH, p30 is negatively charged while the zein NPs are positively charged. The absorption assay revealed that zein NPs:p30 have a mean size of 318.6 ± 18.6 nm, PDI= 0.25 ± 0.14, zeta potential= −16.72 ± 2.65, and AE= 57.9 ± 1.2%. Additionally, a stability analysis of zein NPs:p30 was performed over an incubation period of 28 days (Figure 2). The size remained stable for 2 weeks but increased over time in further time points, reaching mean values of 494.5 ± 14.9 nm at the end of the analysis.

### 3.4. Preparation of Zein NPs:RBD

The RBD protein was adsorbed onto zein NPs following the same protocol used for the p30 peptide (1:2 mass ratio). However, the adsorption process for RBD was performed at pH 5.5 as the theoretical isoelectric point of RBD is 8.9. This pH ensures that the RBD protein remains positively charged, facilitating effective electrostatic interactions with the negatively charged zein NPs. The successful adsorption of RBD onto zein NPs was confirmed by determining the AE, which was found to be 67.9%.

### 3.5. Morphological Characterization by TEM

**Figure 3:**
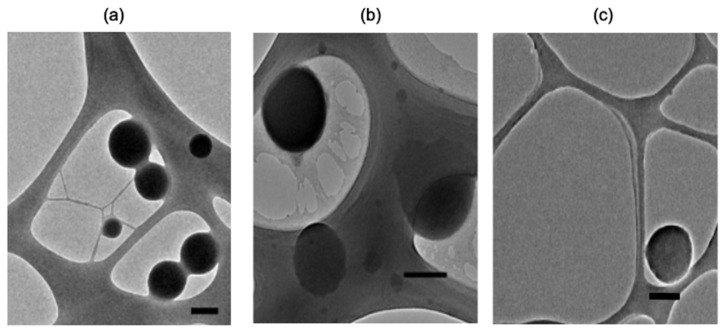
TEM images of representative samples of (**a**) zein NPs, (**b**) zein NPs:BSA, and (**c**) zein NPs:p30 (Scale bar: 200 nm).

TEM imagining of representative samples demonstrated spherical morphology of either zein NPs, zein NPs:BSA, and zein NPs:p30 (Figure 3). TEM images of zein NPs:BSA revealed a layer over the particle surface, suggesting the presence of BSA adsorbed on the surface. Additionally, the diameter was measured using micrographs and the analysis revealed a mean diameter of zein NPs, zein NPs:BSA, and zein NPs:p30 of 152.9 ± 32.6 nm, 241.8 ± 76.2 nm, and 307 ± 63.6 nm, respectively.

### 3.6. Cytotoxicity of ZNPs

**Figure 4:**
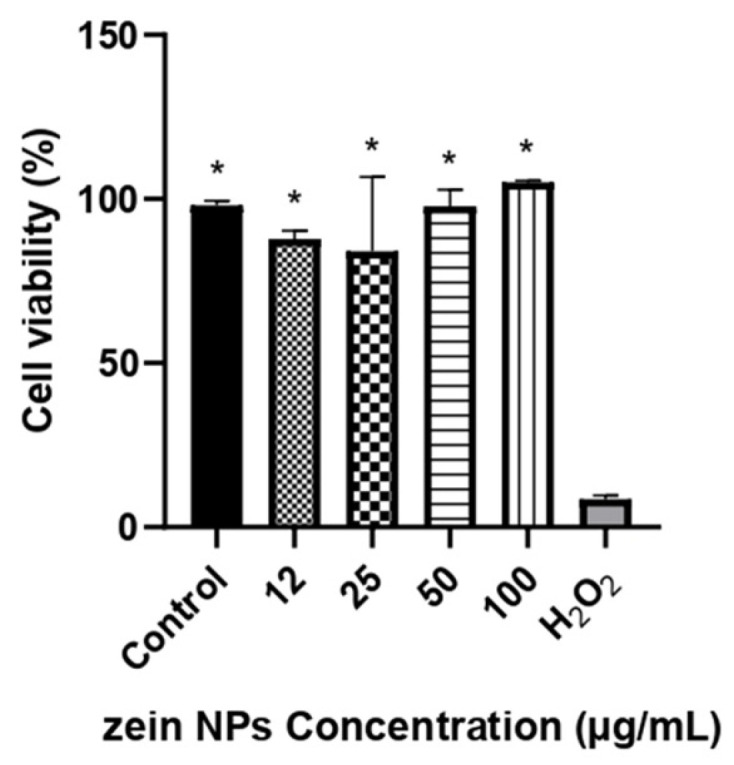
Cell viability in Vero cells following treatment with different concentrations of ZNPs. The asterisks denote significant differences respect the cells treated with H_2_O_2_ (*p* < 0.05). Control cells were treated with the vehicle alone.

Cytotoxicity of bare zein NPs was evaluated in Vero cell line by a resazurin assay in a range of 12–100 µg/mL. The results of the cellular viability are illustrated in Figure 4. After 24 h of incubation with the treatments, the viability was not affected by any of the concentrations tested, with cellular viability values above 84%, which are significative different compared to the control treated with H_2_O_2_ (*p* < 0.05).

### 3.7. Immunogenicity Assay for Zein NPs:p30 

**Figure 5:**
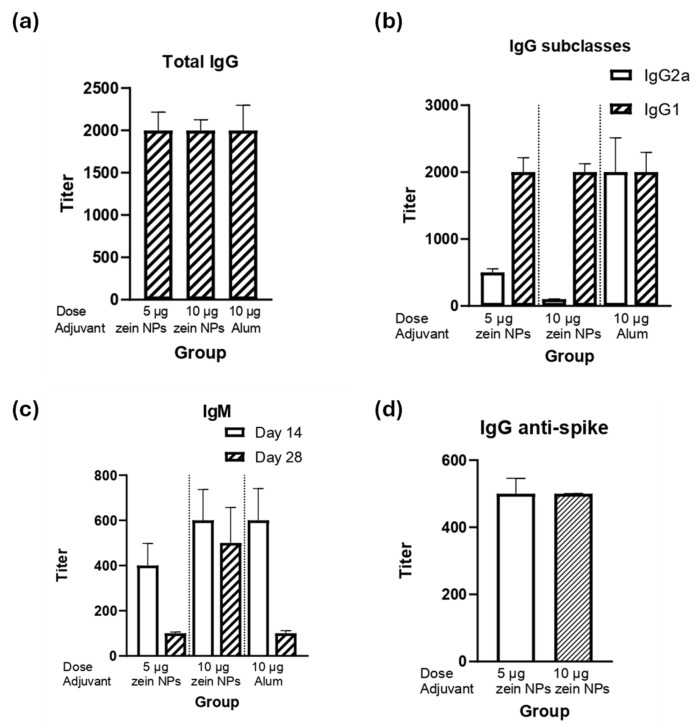
Humoral response induced by the zein NPs:p30 formulation. Mice groups (*n* = 4) were s.c. injected on days 0, 14, and 28 with 5 µg of p30 adsorbed onto zein NPs, 10 µg of p30 adsorbed onto zein NPs, 10 µg of p30 with alum, or the vehicle alone. Blood samples were collected on day 28 to measure serum antibody levels by ELISA. The ELISA plates were coated with p30 peptide to determine total IgG (**a**), IgG subclasses (**b**), and IgM (**c**); or with RBD from spike protein for total IgG determination (**d**). Mean values ± SD are presented.

The ability of the zein NPs:p30 to elicit humoral responses was assessed by performing a mice immunization assay. BALB/c mice were immunized subcutaneously with either 5 or 10 µg of the p30 antigen adsorbed onto zein NPs. The immunogenicity in terms of humoral responses was compared with the vehicle alone as a negative control group or a group immunized with 10 µg of p30 peptide with alum as an adjuvanted positive control group. After the second immunization, the data analysis for total IgG levels demonstrated that groups immunized with either 5 or 10 µg of p30 in zein NPs:p30 had comparable titers respect the positive control group, suggesting that zein NPs act as adjuvants in equal manner as alum (Figure 5a).

The analysis of the IgG subclasses revealed that the IgG1 subclass predominates, which suggests that a Th2-bias (humoral) response is induced (Figure 5b). Interestingly, both groups elicited the same IgG1 titers as the adjuvanted group. Furthermore, as expected, IgM antibodies were elicited upon the first immunization (day 14) and decreased with time (Figure 5c).

Since the p30 peptide is part of the spike protein of SARS-CoV-2, detection of IgG antibodies against the full spike protein was conducted, observing that the group immunized with zein NPs:p30 had antibodies capable to bind the full-length spike protein (Figure 5d).

### 3.8. Immunogenicity Assay for Zein NPs:RBD

**Figure 6:**
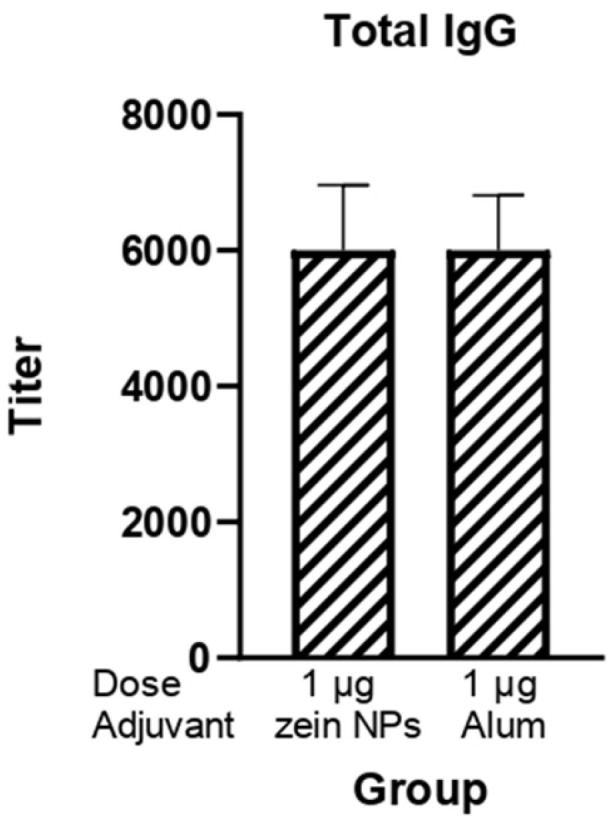
Humoral response induced by the zein NPs:RBD formulation. Mice groups (*n* = 3) were s.c.-injected on days 0, 14, and 21 with 1 µg of RBD protein adsorbed onto zein NPs, 1 µg of RBD protein with alum or the vehicle alone. Blood samples were collected on day 28 to measure serum anti-RBD IgG antibody levels by ELISA. Mean values ± SD are presented.

The potential of zein NPs as effective carriers able to enhance the immune response against the RBD protein was also investigated given its relevance for the induction of neutralizing antibodies against SARS-CoV-2. BALB/c mice were subcutaneously immunized with 1 µg of RBD protein adsorbed onto zein NPs. The immunogenicity of this formulation was systematically compared to a control group that received alum as an adjuvant, which is widely recognized for its ability to enhance immune responses (Figure 6). Following the third immunization, a comprehensive analysis of total IgG levels in serum samples from the immunized mice was conducted. The results demonstrated that the groups receiving 1 µg of RBD adsorbed onto zein NPs elicited total IgG antibody titers comparable to those observed in the group adjuvanted with alum.

## 4. Discussion

In this study we establish a simple method for zein NP synthesis and its use to formulate a vaccine prototype using SARS-CoV-2 antigens as a case of study. The established methods comprised zein NPs synthesis followed by passive adsorption of the antigen, the characterization of the obtained particles, and its stabilization in a physiological buffer that allows its use for immunization.

The most reported methods for zein NP synthesis include nanoprecipitation, liquid–liquid dispersion, phase separation, and electrospraying [30]. In this report, the synthesis of zein NPs was based on the precipitation of zein dispersed in an aqueous alcohol phase, which was added to an aqueous phase, causing a supersaturation of zein as long as ethanol concentration is reduced, leading to precipitation-mediated zein NP formation [27,31]. The order of phase addition impacts the size and aggregation of the particles: larger particle sizes and increased aggregation occur when the aqueous phase is added to the organic phase, as previously reported [27,32]. An initial exploration of this aspect revealed that adding the organic phase to the aqueous phase led to smaller particles and a low PDI, while the opposite order led to larger and highly polydisperse particles. The performance of the method was improved by introducing the use of a surfactant in the aqueous phase to avoid particle aggregation, leading to a highly stable particle suspension; therefore, this approach was adopted for further optimization.

**Figure 7:**
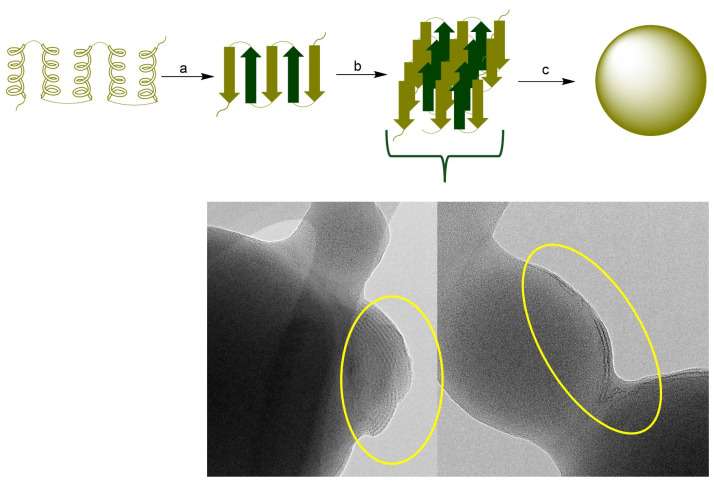
(**Top**): (a) Conformational transition from a-helix to β-sheet, (b) Packing of b-sheets (c) nanoparticle formation. (**Bottom**): TEM image of the surface of zein NPs. The circle presents the evidence of the formation of β-sheets packed.

Zein protein composition consists of the mixture of four main proteins that vary in amino acid sequences, molecular weight (MW), and solubility. These include α-zein (MW, 24–27 kDa; 75–80% of total protein), β-zein (17–18 kDa, 10–15%), γ-zein, and δ-zein (27 kDa, and 10 kDa, respectively; representing 5–10%). The large proportion (>50%) of non-polar amino acids among these proteins, such as leucine, proline, alanine, and phenylalanine, makes them water insoluble. The hydrophobicity of zein favors the ease formation of zein NPs by a mechanism that involves the hydrophobic contraction and nucleation of induced by changes in the polarity of the solvent system. The tertiary structure of zein comprises α-helix and β-sheet structures. In an alcohol–water solution, zein undergoes a structural transition from α-helix to β-sheet. The β conformation of zein forms laminar structures (β-sheets) due to the hydrogen bond formation. It has been demonstrated that maximal change in the globular structure of zein promotes nanoparticle formation. Additionally, β-sheet lattices form a striped pattern with aligned lattice line [33,34,35]. The diagram in Figure 7 represents the general mechanism of zein NPs formation. The TEM micrograph of zein NPs evidences the formation of β-sheets packed side by side forming a long ribbon structure.

Different solvents have been used during zein NP synthesis, including methanol, isopropanol, and ethanol [36]. In the present study, we initially evaluated these solvents and observed that using 80% ethanol as zein solvent resulted in the smallest particle size (mean value of 207 ± 36.7 nm). Therefore, this solvent was selected for further experiments.

Another key factor during zein NP synthesis is zein concentration, since it has a significant impact on the behavior of the colloidal system because the concentration of the particles under formation is directly related with the rheological properties such as viscosity, flow behavior, and stability. Our study confirmed this effect as zein NPs with larger size and high PDI were obtained when a higher initial zein concentration was used. The increase in zein concentration increases the viscosity of the dispersion, which directly affects the nucleation process promoting the formation of larger particles. Moreover, the effect of particle concentration on AE was observed, revealing an anomalous phenomenon in which the adsorption decreases as the particle concentration increases [37].

Optimizing nanoparticle synthesis represents several challenges not only at the synthesis phase but also the achievement of a stable colloidal system with minimum aggregation rates. To pursue this goal, the use of surfactants during nanoparticle synthesis is an effective approach. Surfactants are organic compounds having functional groups with different polarities, one of them with affinity for polar phases and another attracted to nonpolar phases [38]. Due to their intrinsic hydrophobicity, zein particles tend to lose physical stability and aggregate. The stabilizing effect of the addition of Tween 20 was previously studied during the synthesis of zein NPs, revealing that using Tween 20 at 0.2 g/L is critical for the formation of small and stable zein NPs and suggested that the hydrophobic moieties of zein molecules interact with the alkyl chains of Tween-20 molecules [39].

**Figure 8:**
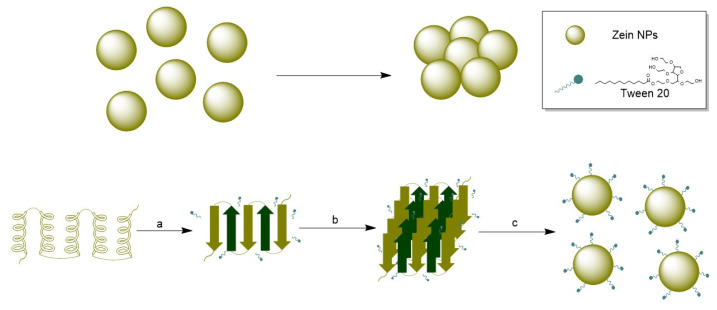
(**Top**): Agglomeration of zein NPs in absence of a stabilizer. (**Bottom**): (a) Conformational transition from α-helix to β-sheet, (b) Packing of β-sheets in presence of Tween 20, (c) nanoparticle formation and stabilization by Tween 20.

Since Tween 20 was introduced during the formation of zein NPs, this small-molecule surfactant may preferentially bind to zein, resulting in the formation of a zein/Tween 20 complex that prevents protein aggregation. Wang and Chu (2018) investigated the role of surfactants in the formation of zein NPs [39]. They observed that at low concentrations of Tween 20 (0–0.2 g/L), the alkyl chains of Tween 20 molecules predominantly interact with zein molecules through hydrophobic interactions, leading to the formation of zein/Tween 20 complexes. This complexation promotes the unfolding of the zein protein, resulting in a decrease in α-helix content. Additionally, hydrogen bonds may form between polar amino acids in zein and the ethylene oxide groups in Tween 20, which reduces hydrophobic attractions and increases steric repulsion between zein/Tween 20 complexes. The hydrophilic head groups of Tween 20 that extend into the aqueous phase may enhance the hydrophilicity of the zein/Tween 20 particles (Figure 8) [39,40]. Our data align with these findings, as the incorporation of Tween 20 resulted in a highly stable suspension of the synthesized zein NPs.

Size is a critical trait for nanosystems since it determines several properties responsible for their efficacy as delivery agents. For instance, surface area is critical to ensure efficient loading with the cargo. Moreover, to penetrate cells, a low particle size, in the range of 100–300 nm, is required. In particular, for vaccines, the uptake of the binanoconjugate by APCs must be performed to process the antigen and present it to lymphocytes. The size obtained after standardizing the method was comparable to that reported in other studies [31,41].

**Figure 9:**
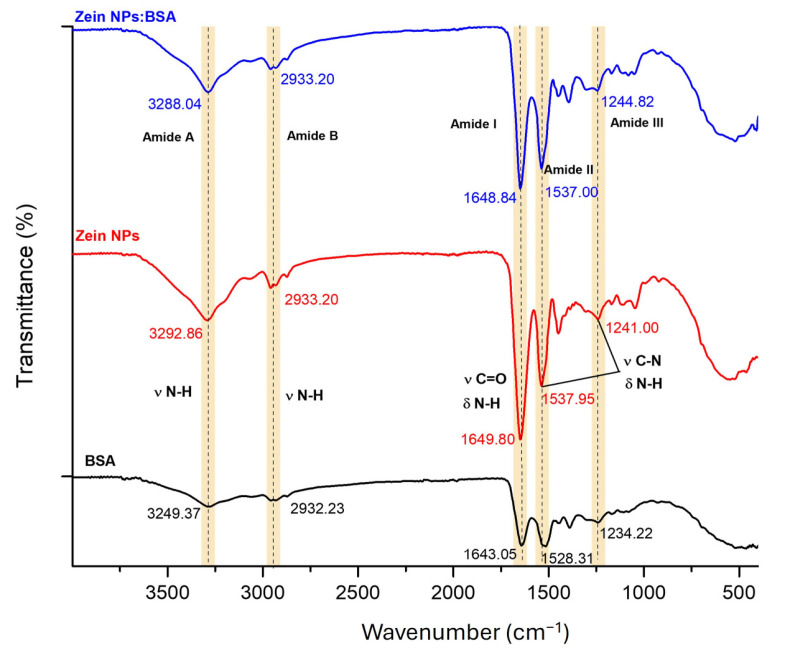
FT-IR spectroscopy analysis of bare zein NPs, BSA alone, and zein NPs:BSA (Dashed lines allow to better visualize changes in vibration frequencies).

To evaluate the capacity of zein NPs for loading antigens via physical adsorption, BSA was selected as a model antigen. Fourier-transform infrared (FT-IR) spectroscopy was employed to compare the zein NPs before and after BSA adsorption, aiming to investigate the interactions between the two proteins (Figure 9). Characteristic bands associated with natural proteins were observed at 3249.37 and 3292.86 cm^−1^ (Amide A), 2932.23 and 2933.20 cm^−1^ (Amide B), 1643.05 and 1649.80 cm^−1^ (Amide I), 1528.31 and 1537.95 cm^−1^ (Amide II), and 1234.22 and 1241.00 cm^−1^ (Amide III) for BSA and zein NPs, respectively. The Amide I region, which is attributed to the stretching vibration of υ N-H bonds for zein NPs:BSA, exhibited a slight shift (from 3292.86 to 3288.04 cm^−1^) following BSA adsorption, suggesting the formation of hydrogen bond interactions between zein NPs and BSA. Additionally, minor changes in the intensity and position of peaks in the Amide II and Amide III regions, corresponding to υ C=O and υ C-N groups, were observed. These alterations are likely due to non-covalent interactions, including electrostatic interactions and hydrogen bonding effects between zein NPs and BSA [42].

**Figure 10:**
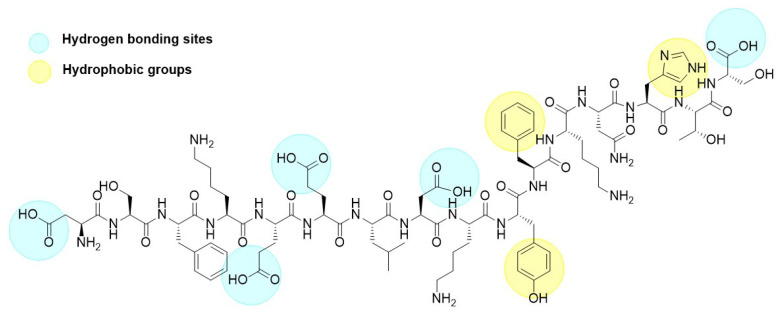
Chemical structure of the p30 peptide.

The adsorption of p30 onto zein NPs was conducted at a mass ratio of 1:2. Considering that the theoretical isoelectric point of p30 is 5.48 and the PZC for zein is 6.2, a 20 mM Tris buffer at pH 5.8 was used for the adsorption process. At this pH, it is assumed that p30 will carry a negative charge while the zein NPs will be positively charged. The adsorption of p30 onto zein NPs was confirmed by observing changes in the hydrodynamic diameter of the nanoparticles. The mean size of the resulting zein NPs:p30 bio increased to 318.6 ± 18.3 nm, which is considered suitable for antigen delivery to APCs. Additionally, the PDI of 0.25 ± 0.14 indicates a monodisperse size distribution within the suspension. TEM images corroborated these findings, revealing a spherical morphology for the zein NPs:p30, suggesting efficient adsorption of p30 onto the surface of the zein NPs. Regarding zeta potential measurements, bare zein NPs in the adsorption buffer exhibited mean values of −2.98 ± 4.11 mV, which changed upon adsorption of p30 to −16.72 ± 2.65 mV. This change was expected due to the negatively charged nature of the adsorbed antigen, which resulted in a more negative zeta potential. The increase in surface negative charges was directly related to the presence of the p30 peptide on the surface of the zein NPs. The presence of aromatic groups in the peptide structure enhances hydrophobic interactions with zein NPs, while carboxylic acid groups promote hydrogen bond formation (Figure 10). Together with electrostatic interactions between p30 and zein NPs, these factors contribute to the generation of a stable compound.

One of the main challenges for the synthesis of nanovaccines is ensuring that the physicochemical conditions during synthesis and functionalization are compatible with biological applications. For instance, the pH at which optimal synthesis takes place must ensure the stability of the nanoparticle and, subsequently, for its administration to the test organism. Therefore, this parameter should fall in the range of physiologic fluids, that is an approximate value of 7.6. Buffers capable of maintaining a pH range between 5–8 values include acetate, citrate, histidine, phosphate, and Tris [43,44]. To determine the optimal buffer for our method, zein NPs were resuspended in two different buffer solutions, previously reported for both dispersion of zein NPs and loading with specific drugs: 20 mM Tris and citrate buffer, both at pH 7.4 [45]. Interestingly, Tris buffer resulted in a better stability compared with citrate buffer, and thus, the former was selected for vaccine formulation.

In this study, the p30 peptide was utilized as the target antigen to assess its immunogenicity. The p30 peptide, derived from the spike protein of SARS-CoV-2, is a key target in vaccine development and has been identified as a B-cell epitope, which are essential for inducing humoral immune responses that may protect against infectious diseases.

In this context, our study demonstrated that immunization with either 5 or 10 µg of zein NPs:p30 induced similar levels of total IgG antibody titers as the alum-adjuvanted group, suggesting that zein NPs exhibit adjuvant properties. The same effect was observed with zein NPs:RBD. These findings underscore the adjuvant properties of zein NPs, indicating that they can effectively enhance the immunogenicity of the RBD protein. Collectively, these findings suggest that zein NPs serve as efficient delivery vehicles/adjuvants for vaccine development. This study aligns with previous research showing that organic nanoparticles exert adjuvanticity [46,47]. Additionally, the analysis of IgG subclasses revealed a marked predominance of IgG1 over IgG2a, indicating a Th2-type immune response. IgG subclass analysis provides insight into the nature of the immune response, with the dominant IgG1 production signaling a Th2-mediated humoral response. This response is crucial for effective adaptive immunity, as it mediates antibody-dependent protection, facilitates long-term protection through memory B cells, and ensures an effective defense against several pathogens. Therefore, vaccines that can induce a strong Th2 response are often highly effective in preventing infections that are primarily targeted by humoral immunity [48,49].

Interestingly, this method offers a simple and efficient approach for modifying the system to adsorb any antigen by adjusting the pH to a level where the antigen acquires a positive charge, in conjunction with an appropriate adsorption buffer. However, it is important to note that each antigen exhibits unique characteristics, which may influence its adsorption behavior and efficiency.

## 5. Conclusions

Zein NPs were successfully synthesized as nanocarriers for the p30 and RBD antigens derived from SARS-CoV-2. The results confirmed that zein NPs exhibit adjuvant activity, enhancing humoral immune responses. The antigen-conjugated zein NPs hold significant promise as a versatile nanovaccine platform, offering a rapid, environmentally friendly and straightforward production method. This technology could facilitate the swift development of nanovaccines, providing an effective strategy for responding to emerging infectious diseases by promoting robust humoral immunity.

## Figures and Tables

**Table 1:** Physicochemical characterization of zein NPs:BSA at different mass ratio adsorption (*w*:*w*). Data expressed as mean ± SD, *n* = 3.

Formulation	Mass Ratio (*w*:*w*)	Size (nm)	PDI	Zeta Potential (mV)	AE (%)
zein NPs:BSA	1:0.5	355.7 ± 58.6	0.39 ± 0.08	−11.94 ± 1.27	49.8 ± 5.6
	1:1	372.8 ± 41.7	0.38 ± 0.08	−14.02 ± 4.36	55.4 ± 1.5
	1:2	424.3 ± 36.3	0.27 ± 0.109	−17.89 ± 4.78	63.8 ± 2.1

## Data Availability

The original contributions presented in the study are included in the article; further inquiries can be directed to the corresponding author/s.

## Supplementary Materials

The following supporting information can be downloaded at: https://www.mdpi.com/article/10.3390/vaccines13020139/s1, Table S1: Summary results of ZNP synthesis characterized by DLS under (a) the solvent effect, (b) concentration effect, (c) surfactant addition, and (d) resuspension buffer. Indicated values correspond to mean values ± SD.

## Abbreviations

The following abbreviations are used in this manuscript:

AE	Adsorption efficacy
ANOVA	Analysis of variance
APC	Antigen presenting cells
BSA	Bovine serum albumin
COVID-19	Coronavirus disease 2019
DLS	Dynamic light scattering
FDA	Food and Drug Administration
GALT	Gut-associated lymphoid tissue
GRAS	Generally recognized as safe
MWCO	Molecular weight cut-off
NPs	Nanoparticles
PBS	Phosphate-buffered saline
PDI	Polydispersity index
PLGA	Poly(lactic-co-glycolic acid)
RBD	Receptor binding domain
SARS-CoV-2	Severe acute respiratory syndrome coronavirus 2
TEM	Transmission electron microscopy

## References

[1] Lozano D., Larraga V., Vallet-Regí M., Manzano M. (2023). An Overview of the Use of Nanoparticles in Vaccine Development. Nanomater.

[2] Moghadas S.M., Vilches T.N., Zhang K., Wells C.R., Shoukat A., Singer B.H., Meyers L.A., Neuzil K.M., Langley J.M., Fitzpatrick M.C. (2021). The impact of vaccination on COVID-19 outbreaks in the United States. Clin. Infect. Dis..

[3] Anderson E.J., Rouphael N.G., Widge A.T., Jackson L.A., Roberts P.C., Makhene M., Chappell J.D., Denison M.R., Stevens L.J., Pruijssers A.J. (2020). mRNA-1273 Study Group. Safety and Immunogenicity of SARS-CoV-2 mRNA-1273 Vaccine in Older Adults. N. Engl. J. Med..

[4] Khan Y., Sadia H., Ali Shah S.Z., Khan M.N., Shah A.A., Ullah N., Ullah M.F., Bibi H., Bafakeeh O.T., Khedher N.B. (2022). Classification, Synthetic, and Characterization Approaches to Nanoparticles, and Their Applications in Various Fields of Nanotechnology: A Review. Catalysts.

[5] Joudeh N., Linke D. (2022). Nanoparticle Classification, Physicochemical Properties, Characterization, and Applications: A Comprehensive Review for Biologists. J. Nanobiotechnol..

[6] Patil V., Patel A. (2020). Biodegradable Nanoparticles: A Recent Approach and Applications. Curr. Drug Targets.

[7] Biswas S., Chattopadhyay M., Sen K.K., Saha M.K. (2015). Development and Characterization of Alginate Coated Low Molecular Weight Chitosan Nanoparticles as New Carriers for Oral Vaccine Delivery in Mice. Carbohydr. Polym..

[8] Tzeng S.Y., McHugh K.J., Behrens A.M., Rose S., Sugarman J.L., Ferber S., Langer R., Jaklenec A. (2018). Stabilized Single-Injection Inactivated Polio Vaccine Elicits a Strong Neutralizing Immune Response. Proc. Natl. Acad. Sci. USA..

[9] Bovier P.A. (2008). Epaxal^®^: A Virosomal Vaccine to Prevent Hepatitis A Infection. Expert. Rev. Vaccines.

[10] Mischler R., Metcalfe I.C. (2002). Inflexal V: A Trivalent Virosome Subunit Influenza Vaccine: Production. Vaccine.

[11] Manish M., Rahi A., Kaur M., Bhatnagar R., Singh S. (2013). A Single-Dose PLGA Encapsulated Protective Antigen Domain 4 Nanoformulation Protects Mice Against Bacillus anthracis Spore Challenge. PLoS ONE.

[12] Luo Y., Wang Q., Zhang Y. (2020). Biopolymer-Based Nanotechnology Approaches to Deliver Bioactive Compounds for Food Applications: A Perspective on the Past, Present, and Future. J. Agric. Food Chem..

[13] Oleandro E., Stanzione M., Buonocore G.G., Lavorgna M. (2024). Zein-Based Nanoparticles as Active Platforms for Sustainable Applications: Recent Advances and Perspectives. Nanomaterials.

[14] Zhang Y., Cui L., Li F., Shi N., Li C., Yu X., Chen Y., Kong W. (2016). Design, Fabrication and Biomedical Applications of Zein-Based Nano/Micro-Carrier Systems. Int. J. Pharm..

[15] Paliwal R., Palakurthi S. (2014). Zein in Controlled Drug Delivery and Tissue Engineering. J. Control. Release.

[16] Luo Y., Teng Z., Wang Q. (2012). Development of Zein Nanoparticles Coated with Carboxymethyl Chitosan for Encapsulation and Controlled Release of Vitamin D3. J. Agric. Food Chem..

[17] Peñalva R., Esparza I., González-Navarro C.J., Quincoces G., Peñuelas I., Irache J.M. (2015). Zein Nanoparticles for Oral Folic Acid Delivery. J. Drug Deliv. Sci. Technol..

[18] Dong F., Dong X., Zhou L., Xiao H., Ho P.-Y., Wong M.-S., Wang Y. (2016). Doxorubicin-Loaded Biodegradable Self-Assembly Zein Nanoparticle and Its Anti-Cancer Effect: Preparation, In Vitro Evaluation, and Cellular Uptake. Colloids Surf. B Biointerfaces.

[19] Lai L., Guo H. (2011). Preparation of New 5-Fluorouracil-Loaded Zein Nanoparticles for Liver Targeting. Int. J. Pharm..

[20] Zhang Q., Li D., Guan S., Liu D., Wang J., Xing G., Yue L., Cai D. (2022). Tumor-Targeted Delivery of Honokiol via Polysialic Acid Modified Zein Nanoparticles Prevents Breast Cancer Progression and Metastasis. Int. J. Biol. Macromol..

[21] Yang Y., Jia Y., Zhang M., Luo Y., Zhang Z., Wu W., Yuan L. (2024). Synthesis, Characterization, Antioxidant and Bacteriostasis in Preservation of Isoorientin Loaded Zein/GA Nanoparticles. Food Chem. X.

[22] Li S., Wang X., Li W., Yuan G., Pan Y., Chen H. (2016). Preparation and Characterization of a Novel Conformed Bipolymer Paclitaxel-Nanoparticle Using Tea Polysaccharides and Zein. Carbohydr. Polym..

[23] Huang X., Liu Y., Zou Y., Liang X., Peng Y., McClements D.J., Hu K. (2019). Encapsulation of Resveratrol in Zein/Pectin Core–Shell Nanoparticles: Stability, Bioaccessibility, and Antioxidant Capacity after Simulated Gastrointestinal Digestion. Food Hydrocoll..

[24] Feng S., Sun Y., Wang D., Sun P., Shao P. (2020). Effect of Adjusting pH and Chondroitin Sulfate on the Formation of Curcumin-Zein Nanoparticles: Synthesis, Characterization and Morphology. Carbohydr. Polym..

[25] Bidyarani N., Kumar U. (2019). Synthesis of Rotenone Loaded Zein Nano-Formulation for Plant Protection Against Pathogenic Microbes. RSC Adv..

[26] Wang G., Han J., Meng X., Kang S.S., Liu X., Sun Y.E., Luo Q., Ye K. (2023). Zein-Based Nanoparticles Improve the Therapeutic Efficacy of a TrkB Agonist Toward Alzheimer’s Disease. ACS Chem. Neurosci..

[27] Meewan J., Somani S., Almowalad J., Laskar P., Mullin M., MacKenzie G., Khadke S., Perrie Y., Dufès C. (2022). Preparation of Zein-Based Nanoparticles: Nanoprecipitation Versus Microfluidic-Assisted Manufacture, Effects of PEGylation on Nanoparticle Characteristics and Cellular Uptake by Melanoma Cells. Int. J. Nanomed..

[28] Podaralla S., Perumal O. (2012). Influence of Formulation Factors on the Preparation of Zein Nanoparticles. AAPS Pharm. Sci. Tech..

[29] Zhang W., Zhang J., Hu F., Wang W., Du Z., Ke Y., Ma Q., Mou X., Lu J., Yang Z. (2024). Active Dual-Protein Coating Assisted by Stepwise Protein-Protein Interactions Assembly Reduces Thrombosis and Infection. Adv. Sci. (Weinh.).

[30] Pascoli M., de Lima R., Fraceto L.F. (2018). Zein Nanoparticles and Strategies to Improve Colloidal Stability: A Mini-Review. Front. Chem..

[31] Nunes R., Baião A., Monteiro D., das Neves J., Sarmento B. (2020). Zein Nanoparticles as Low-Cost, Safe, and Effective Carriers to Improve the Oral Bioavailability of Resveratrol. Drug Deliv. Transl. Res..

[32] Khayata N., Abdelwahed W., Chehna M.F., Charcosset C., Fessi H. (2012). Preparation of Vitamin E Loaded Nanocapsules by the Nanoprecipitation Method: From Laboratory Scale to Large Scale Using a Membrane Contactor. Int. J. Pharm..

[33] Tapia-Hernández J.A., Rodríguez-Felix F., Juárez-Onofre J.E., Ruiz-Cruz S., Robles-García M.A., Borboa-Flores J., Wong-Corral F.J., Cinco-Moroyoqui F.J., Castro-Enríquez D.D., Del-Toro-Sánchez C.L. (2018). Zein-Polysaccharide Nanoparticles as Matrices for Antioxidant Compounds: A Strategy for Prevention of Chronic Degenerative Diseases. Food Res. Int..

[34] Hu K., McClements D.J. (2014). Fabrication of Surfactant-Stabilized Zein Nanoparticles: A pH Modulated Antisolvent Precipitation Method. Food Res. Int..

[35] Wang Y., Padua G.W. (2012). Nanoscale characterization of zein self-assembly. Langmuir.

[36] Yue Y., Geng S., Shi Y., Liang G., Wang J., Liu B. (2019). Interaction Mechanism of Flavonoids and Zein in Ethanol-Water Solution Based on 3D-QSAR and Spectrofluorimetry. Food Chem..

[37] Utomo H.D., Hunter K.A. (2010). Particle Concentration Effect: Adsorption of Divalent Metal Ions on Coffee Grounds. Bioresour. Technol..

[38] Shaban S.M., Khan J., Kim D.H. (2020). Surfactants: Recent Advances and Their Applications. Compos. Commun..

[39] Wang X., Chu X. (2018). Role of Surfactant in the Formation of Zein/Tween-20 Nanoparticles Studied by Fluorescence and Circular Dichroism. Colloids Surf. A Physicochem. Eng. Asp..

[40] Lee H.J., McAuley A., Schilke K.F., McGuire J. (2011). Molecular Origins of Surfactant-Mediated Stabilization of Protein Drugs. Adv. Drug Deliv. Rev..

[41] Zhong Q., Jin M. (2009). Nanoscalar Structures of Spray-Dried Zein Microcapsules and In Vitro Release Kinetics of the Encapsulated Lysozyme as Affected by Formulations. J. Agric. Food Chem..

[42] Zhang Z., Li R., Tang H., Li J.-X. (2023). Interactions and Characterization of Zein-BSA Nanoparticles: Multi-Spectral Analysis and Molecular Simulations. J. Mol. Liq..

[43] Usach I., Martinez R., Festini T., Peris J.E. (2019). Subcutaneous Injection of Drugs: Literature Review of Factors Influencing Pain Sensation at the Injection Site. Adv. Ther..

[44] Ascendia P. Top Considerations for Formulations of Injectables. Ascendia Pharma 2021. https://ascendiapharma.com/newsroom/2021/08/11/top-considerations-formulations-injectables.

[45] Weissmueller N.T., Lu H.D., Hurley A., Prud’homme R.K. (2016). Nanocarriers from GRAS Zein Proteins to Encapsulate Hydrophobic Actives. Biomacromolecules.

[46] Liu Z., Zhou C., Qin Y., Wang Z., Wang L., Wei X., Zhou Y., Li Q., Zhou H., Wang W. (2017). Coordinating Antigen Cytosolic Delivery and Danger Signaling to Program Potent Cross-Priming by Micelle-Based Nanovaccine. Cell Discov..

[47] Luo Z., Li P., Deng J., Gao N., Zhang Y., Pan H., Liu L., Wang C., Cai L., Ma Y. (2013). Cationic Polypeptide Micelle-Based Antigen Delivery System: A Simple and Robust Adjuvant to Improve Vaccine Efficacy. J. Control Release.

[48] Bettini E., Locci M. (2021). SARS-CoV-2 mRNA Vaccines: Immunological Mechanism and Beyond. Vaccines.

[49] Chen S., Guan F., Candotti F., Benlagha K., Camara N.O.S., Herrada A.A., James L.K., Lei J., Miller H., Kubo M. (2022). The Role of B Cells in COVID-19 Infection and Vaccination. Front. Immunol..

