# Supplementary material for: Evaluation of Zein Nanoparticles as Delivery Agents of SARS-CoV-2 Antigens
Source: Vaccines (Basel). 2025 Jan 28;13(2):139. doi: 10.3390/vaccines13020139 (PMC11860552; doi:10.3390/vaccines13020139)
Supplement: Supplementary file 1 [file vaccines-13-00139-s001.zip › vaccines-3411041-supplementary.pdf]

Table S1: Summary results of ZNP synthesis characterized by DLS under (a) the solvent effect (b) concentration effect (c) surfactant addition, and (d) resuspension buffer. Indicated values correspond to mean values ± SD.

(a)

Solvent	Size (nm)	PDI	Zeta Potential (mV)
Methanol	256.24 ± 23.2	0.185 ± 0.066	-41.35 ± 14.8
Isopropanol	639.34 ± 74.3	0.208 ± 0.047	-39.39 ± 3.6
Ethanol	207 ± 36.7	0.188 ± 0.010	-24.8 ± 10.4

(b)

Zein concentration (mg/mL)	Size (nm)	PDI	Zeta Potential (mV)
5	357.9 ± 57.70	0.34 ± 0.14	-13.7 ± 2.2
2.5	276.35 ± 96.12	0.32 ± 0.04	-10.84 ± 1.07
0.5	220 ± 10	0.13 ± 0.15	-8.9 ± 0.89

(c)

Surfactant	Days	Size (nm)	PDI	Zeta Potential (mV)
Tween20	0	220 ± 10	0.13 ± 0.15	-9.8 ± 1.45
	7	228.5 ± 5	0.17 ± 0.08	-8.9 ± 2.65
	14	231 ± 8	0.18 ± 0.10	-9.2 ± 1.8
	28	232 ± 9.2	0.14 ± 0.09	-11.3 ± 0.5
No	0	228.8 ± 15	0.11 ± 0.02	-10.23 ± 1.89
	7	304.7 ± 11.5	0.34 ± 0.11	-5.89 ± 3.89
	14	802.8 ± 15	0.621 ± 0.2	-3.23 ± 1.56
	28	939.2 ± 23.9	0.72 ± 0.18	-2.58 ± 1.79

(d)

Day	Buffer	Size (nm)	PDI	Zeta Potential (mV)
0	Tris	220 ± 10	0.13 ± 0.15	-9.8 ± 1.45
	Citrate	234.9 ± 3.5	0.16 ± 0.09	-8.7 ± 0.89
7	Tris	228.5 ± 5	0.17 ± 0.08	-8.9 ± 2.65
	Citrate	242.2 ± 6.9	0.26 ± 0.15	-9.9 ± 1.89
14	Tris	231 ± 8	0.18 ± 0.10	-9.2 ± 1.8
	Citrate	245 ± 15	0.29 ± 0.12	-5.8 ± 3.5
28	Tris	232 ± 9.2	0.14 ± 0.09	-11.3 ± 0.5
	Citrate	278.2 ± 23.7	0.31 ± 0.05	-6.4 ± 0.8
